# The prevalence of diabetes in Afghanistan: a systematic review and meta-analysis

**DOI:** 10.1186/s12889-021-10993-5

**Published:** 2021-05-17

**Authors:** Sohail Akhtar, Jamal Abdul Nasir, Amara Javed, Mariyam Saleem, Sundas Sajjad, Momna Khan, Abdul Wadood, Khwaja Saeed

**Affiliations:** 1 Department of Statistics, Government College University, Lahore, Pakistan; 2 School of Statistics, Minhaj University, Lahore, Pakistan; 3 Department of Medical Biotechnology, Edinburgh Napier University , Edinburgh, UK; 4 Ministry of Public Health, Afghanistan National Public Health Institute (ANPHI), Kabul, Afghanistan

**Keywords:** Prevalence, Diabetes, Afghanistan, Meta-analysis and systematic review

## Abstract

**Background:**

The aim of this paper is to investigate the prevalence of diabetes and its associated risk factors in Afghanistan through a systematic review and meta–analysis.

**Methods:**

A comprehensive literature search was conducted using EMBASE, PubMed, Web of Sciences, Google Scholar and the Cochrane library, carried out from inception to April 312,020, without language restriction. Meta–analysis was performed using DerSimonian and Laird random-effects models with inverse variance weighting. The existence of publication bias was initially assessed by visual inspection of a funnel plot and then tested by the Egger regression test. Subgroup analyses and meta-regression were used to explore potential sources of heterogeneity. This systematic review was reported by following the PRISMA guidelines and the methodological quality of each included study was evaluated using the STROBE guidelines.

**Results:**

Out of 64 potentially relevant studies, only 06 studies fulfilled the inclusion criteria and were considered for meta-analysis. The pooled prevalence of diabetes in the general population based on population-based studies were 12.13% (95% CI: 8.86–16.24%), based on a pooled sample of 7071 individuals. Results of univariate meta-regression analysis revealed that the prevalence of diabetes increased with mean age, hypertension and obesity. There was no significant association between sex (male vs female), smoking, the methodological quality of included articles or education (illiterate vs literate) and the prevalence of diabetes.

**Conclusions:**

This meta-analysis reports the 12.13% prevalence of diabetes in Afghanistan,with the highest prevalence in Kandahar and the lowest in Balkh province. The main risk factors include increasing age, obesity and hypertension. Community-based care and preventive training programmes are recommended.

**Trial registration:**

This review was registered on PROSPERO (registration number CRD42020172624).

**Supplementary Information:**

The online version contains supplementary material available at 10.1186/s12889-021-10993-5.

## Background

Diabetes is a major public health problem and its frequency is drastically increasing all over the world [1]. The prevalence of type-2 diabetes is rising worldwide as a result of ageing of the population [2], rising levels of overweight and obesity in youth and young adults [3], increasing levels of physical inactivity and poor diet [4]. Globally, it is projected that the diabetes prevalence will increase from 451 million people in the year 2017 to 693 million people by the end of 2045 [5], with 79% of all diabetes cases occurring in low and middle income countries [6]. Worldwide, diabetes prevalence was estimated to be 9% in 2014 among the adult population older than 18 years [1]. It was also reported that 49.7% of individuals living with diabetes are still undiagnosed [7]. The life expectancy of individuals with type-2 diabetes is reduced by approximately 10 years compared with those without the condition, and 80% of type-2 diabetes patients die from cardiovascular complications [7]. More than 60% of the world diabetic population reside in Asian countries, and Asians are more likely to develop diabetes at earlier ages, and with a lower body mass index, than Europeans [8].

Afghanistan is one of the Asian developing countries and facing a growth in the prevalence of diabetes. According to the World Diabetes Foundation estimates, around 1 million people are living with diabetes in Afghanistan and between one and 2 million diabetes cases are undiagnosed [9]. According to the human development index of the United Nations, Afghanistan stands at 168th position out of 189 countries and territories [10]. With limited health care facilities for diabetics, Afghanistan is ill-equipped to cope with this epidemic. Unfortunately, there has been no official nationwide survey to estimate the prevalence of diabetes in Afghanistan. However, a few research studies have been published which report the prevalence of diabetes and its risk factors in different regions of the country. This prevalence in the general population varies among the published studies. The main purpose of this systematic review and meta-analysis is to identify, summarize and estimate the pooled prevalence of diabetes in Afghanistan based on published studies. In addition, we also investigate the associated risk factors of diabetes.

To the best of our knowledge, this is the first systematic review and meta-analysis to estimate the pooled prevalence of diabetes in Afghanistan.

## Methods

### Design

The results were defined using the Preferred Reporting Items for Systematic and Meta-analyses (PRISMA) guidelines [11]. The PRISMA 2009 checklist is attached in supplementary file S1. The protocol of this study was registered with the International Prospective Register of Systematic Reviews (PROSPERO), with protocol registration number CRD42020172624.

### Literature search

A comprehensive literature search was conducted to identify potential articles published on the prevalence of diabetes in Afghanistan. The search was carried out systematically using the following electronic websites: PubMed, Medline, EMBASE, Google Scholar and the Cochrane Library. We considered studies published from inception to April 2020. Using MeSH headings, the terms “Type-2 diabetes”, “Type-II diabetes”, “T2D”, “prevalence”, “Impaired glucose tolerance (IGT)”, “risk factors”, “risk factor”, “glucose intolerance”, “glucose abnormalities”, “non-communicable diseases”, “Afghanistan”, and “Afghan” as well as variations thereof were searched for. Additionally, articles were also searched from reference lists in previously included studies.

### Inclusion and exclusion criteria

The criteria for studies to be included were as follows: (i) articles were published in peer-reviewed journals and reported the prevalence of diabetes; (ii) articles reported population-based or community-based surveys; (iii) articles were published in either English or Pashto.

The criteria used to exclude studies were: (i) irrelevant to diabetes; (ii) not providing clear data; (iii) review articles, case series, or case reports; (vi) relating to the Afghan community living outside of Afghanistan; (v) Articles reported duplicated data (for data published in more than one article) the more up-to-date version was considered and the other articles excluded.

### Data extraction

After selecting the relevant articles, two investigators independently screened the titles and abstracts to consider articles for full text review. The investigators then extracted all the necessary data using a standardized data extraction format in Microsoft Office Excel 2013. Any disagreement between the two investigators was resolved by the third investigator whose decision was regarded as final. The extracted information was: first author surname, year of publication, sex ratio (male/female), age, sample size, the prevalence rate of diabetes, the prevalence of smoking, sampling method, study design and the province in which the study was performed. An extract of these collected data is presented in Table 1, using the Preferred Reporting Items for Systematic and Meta-analyses (PRISMA) guidelines [11].

**Table 1:** Characteristics of 10 studies included in the analysis

Author	design	Year of publication	Sampling Method	Setting	Province	% Female	% Males	% Overweight	% Obesity	% Hypertension	% Illiteracy	% Mouth snuff users	% Physical activity	Age	Smoking	undiagnosed	Prevalence	Methodological Quality
Saeed et al. [12]	CS	2012	Random sampling	Urban	Kabul	66.5	33.5	38.10	31.10	33.14	57	11	31	49.5	13.19	NA	13.18	Good
Saeed [13]	CS	2013	Random sampling	Urban	Nangarhar	60.90	39.1	32.20	27.30	30.5	71.9	10.7	59.2	38.8	6.2	NA	11.8	Good
Saeed et al. [14]	CS	2015	Random sampling	Urban	Kabul	52.60	47.40	NA	NA	32.2	68	28.8	8.7	38.6	8.1058	1.4	9.1	Fair
Saeed [15]	CS	2015	Random sampling	Urban	Kandahar	51.2	48.8	34.3	30	34.3	73.2	16.3	21.3	38.3	9.7	3	22.4	Good
Saeed [16]	CS	2017	Random sampling	Urban	Balkh	53.9	46.1	48.5	15	30.9	59.3	8.3	28.2	41.7	9.9	2.2	9.9	Fair
Saeed [17]	CS	2017	Random sampling	Urban	Herat	52.6	47.4	48	14.5	NA	54	NA	NA	40.5	NA	3.3	9.2	Good

### Methodological quality of included studies

Two authors independently assessed the methodological quality of each of included studies by using Strengthening the Reporting of Observational Studies in Epidemiology (STROBE) guidelines [18]. We categorized the quality of each included study as Good (G) if it scored at least 70% of the points, Fair (F) if it scored 50–69% of the points, and Poor (P) if its points score was less than 50%.

### Statistical analysis

Meta-analysis was performed using the statistical software R version. 3.6.1 [19]. for Microsoft Windows, using two packages (“meta” and “metafor”). Random effects meta-analysis models were used to investigate the pooled prevalence of diabetes using DerSimonian and Laird’s approach with 95% confidence intervals (CIs) [20]. The inverse of the Freeman-Tukey double arcsine transformation was used to stabilize the variance of each study [21]. A forest plot was used to assess visually the prevalence estimates and corresponding 95% confidence intervals (CIs) across included studies. For the evaluation of statistical heterogeneity across studies, the *I*^2^-statistic was used [22]. Heterogeneity was considered as high, moderate, low with *I*^2^ values of 75, 50 and 25% respectively. To identify potential sources of heterogeneity, meta-regression and subgroup analyses were conducted by age, year of publication, hypertension, sex, province, literacy rate, obesity and the methodological quality of the included studies. Publication bias was initially assessed by visual inspection of the funnel plot and then tested by the Egger regression test [23, 24].

## Results

### Literature search

**Fig. 1:**
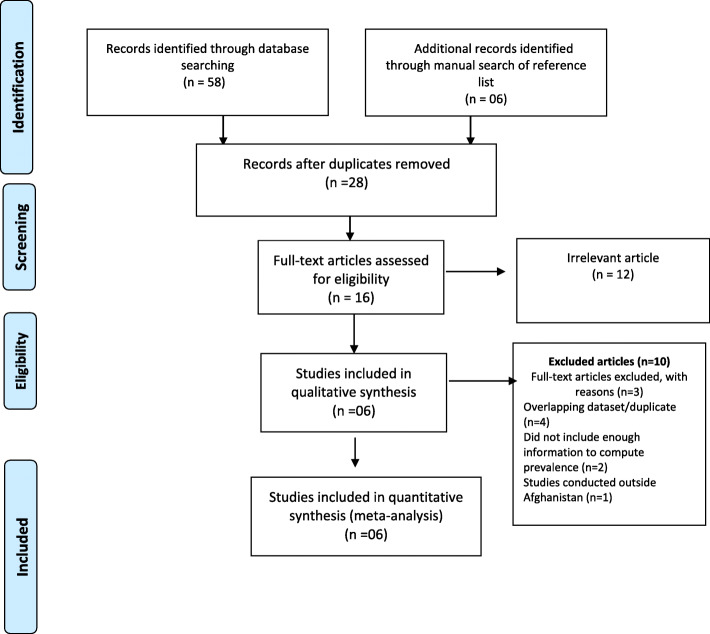
Flow diagram explaining the number of included and excluded articles in the meta-analysis on diabetes in Afghanistan, considered from the PRISMA 2009 guideline [11]

Initially, a total of 64 potential articles was retrieved. Out of these, 36 duplicate articles were removed. After careful review of the titles and abstracts, 12 articles were found irrelevant and then excluded from the process. As the result, only 16 studies were considered for full text reading. Later, 10 studies were excluded after full text reading because they provided no quantitative measure of the prevalence diabetes; overlapped data set; or they were not based in Afghanistan. In the end, only 06 studies were met the inclusion criteria and data were extracted accordingly for the analysis. The flow chart of study selection process is presented in Fig. 1, using the PRISMA flow diagram [11].

### Characteristics of the included studies

The main characteristics of selected studies are summarized in Table 1 [12–17]. All studies used cross-sectional designs and a random sampling procedure. The included studies were published between 2012 and 2017 while the period to which the data relate was from December 2011 to November 2015. The number of subjects per study ranged from 1129 to 1231, for a total of 7071 subjects across studies. Six studies reported the prevalence of diabetes as an outcome of interest. The existence of diabetes was tested with biological measures in all studies. Considering cut-point definitions of diabetes, all studies used a fasting glucose level of 126 mg/dL. Data were collected from five provinces of Afghanistan. Two studies were conducted in Kabul province [12, 14], one study each in Nangarhar province [13], Kandahar [15], Herat [16], and Balkh [17] provinces. The female proportion varied from 51.2% [14] to 66.50% [12]. The average age of participants varied from 38.3 to 49.5 years. The percentage with hypertension ranged from 30.5% [13] to 33.14% [12], based on five studies. The percentage of obese people varied from 15% [16] to 30.1% [12] (six studies). The percentage of illiterate people ranged from 54 to 73.2% (6 studies). The percentage of smokers ranged from 5.1 to 13.7%. The percentage of overweight people ranged from 32.2 to 48.5% (five studies).

### Pooled meta-analysis

**Fig. 2:**
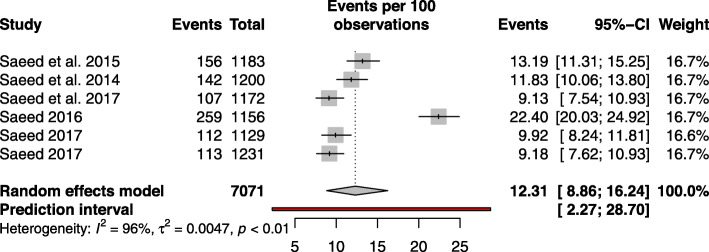
Forest plot of prevalence of diabetes of adult population of Afghanistan

**Fig. 3:**
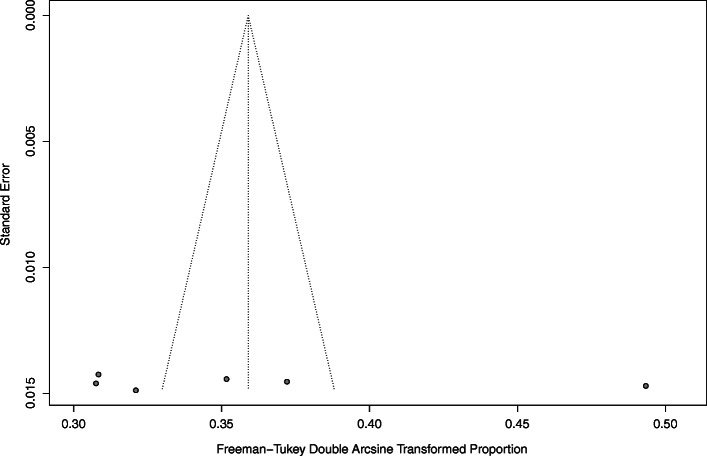
Funnel plot of the prevalence of diabetes in Afghanistan

Statistical analysis of the prevalence of diabetes is described in Table 2. The pooled prevalence of diabetes was 12.13% (95% CI: 8.86–16.24%, *I*^2^ = 99.3%, based on 06 studies) in a total sample of 7071 individuals. The forest plot of the prevalence estimates and their respective 95% confidence intervals (CIs) is presented in Fig. 2. The funnel plot (Fig. 3) showed almost no publication bias which is confirmed by the Egger regression test (*p* = 0.732). Furthermore, the no publication bias was confirmed by ‘Trim and Fill’ sensitivity analysis—as we did not find any hypothetical missing study. The prevalence of undiagnosed diabetes was 9.70% (95% CI: 4.99–15.74, *I*^2^ = 97.5%, based on four studies) with a total sample size of 4697 participants.

**Table 2:** Prevalence of diabetes and its risk factors in the adult population of Afghanistan

Characteristic	Studies	Sample	Cases	Prevalence, % (95%CI)	I^2^, %	Heterogeneity	P-Egger test
Diabetes	6	7071	889	12.31 (8.86–16.24)	0.957	< 0.001	0.5764
Undiagnosed	4	4697	478	9.70 (4.99–15.74)	0.975	< 0.001	0.1267
By Sex							0.0278
Male	6	3976	379	12.07 (8.98–15.55)	0.877	< 0.0001	
Female	6	3095	508	12.56 (8.48–17.29)	0.945	< 0.0001	
By Age							
25–34	4	1922	179	8.45 (3.65–14.97)	0.952	< 0.0001	0.0359
35–44	4	1179	164	13.02 (7.54–19.70)	0.902	< 0.001	
45–54	4	800	140	17.12 (9.42–26.51)	0.907	< 0.001	
55+	4	679	131	19.23 (16.20–22.44)	0.078	0.354	
By Province							0.1263
Kabul	2	2355	263	11.08 (7.42–15.36)	0.50	< 0.001	
Nangarhar	1	1200	536	11.8	–	–	
Balkh	1	1231	113	9.18	–	–	
Herat	1	1129	112	9.92	–	–	
Kandahar	1	1165	261	22.40	–	–

### Heterogeneity and subgroup analysis

The pooled prevalence among females was 12.56% (95% CI: 8.48–17.29), while for males, it was 12.0% (95% CI: 8.98–15.55). When stratified by age, the pooled prevalence in age groups 25–34 years, 35–44 years, 45–54 years and 55 years and over was 8.45% (95% CI: 3.65–14.97), 13.02% (95% CI: 7.54–19.70), 17.12% (95% CI: 9.42–26.51), and 19.23% (95% CI: 16.20–22.44), respectively. The pooled prevalence in the 55 years and over age group was the highest of the four age groups, which shows that the prevalence of diabetes increases with age (Table 2).

When stratified by province, the prevalence of diabetes was highest (22.40%) in Kandahar, compared with 11.08% (95% CI: 7.42–15.36) in Kabul, 11.80% in Nangahar, 9.92% in Herat, and 9.18% in Balkh. The difference in the pooled prevalence of diabetes between males and females was insignificant. There was no significant publication bias in all subgroup analyses.

The result of the univariate meta-regression analysis showed that the prevalence of diabetes increased with a mean age (*β* = 0.0107, 95% CI: 0.0002–0.0208, *p* = 0.0343, *R*^2^ = 42.34%), hypertension (*β* = 0.0377, 95% CI: 0.0034–0.0720, *p* = 0.0311, *R*^2^ = 48.94%) and obesity (*β* = 0.0064, 95% CI: − 0.0007–0. 0135, *p* = 0.070, *R*^2^ = 35.64%). The univariate meta-regression analysis also revealed that there was no significant difference by sex (male vs female), sample size, year of publication, smoking, education (illiterate versus literate) or the methodological quality of included studies.

## Discussion

The purpose of this systematic review was to compile all available data reporting the prevalence of diabetes and related risk factors among the adult population of Afghanistan between 2000 and 2019. The information provided in this systematic review and meta-analysis may contribute to improve public health interventions in the country and therefore contribute to reduce the incidence of diabetes. The results showed that the pooled prevalence of diabetes based on 6 population-based cross-sectional studies was 12.13%, with a total sample of 7071 individuals. Compared with neighbouring countries sharing a similar lifestyle and culture, the pooled prevalence of diabetes in Afghanistan is higher than in Bangladesh (7.8%) [25] and Nepal (8.4%) [26], while lower than in Pakistan (14.7%) [27].

Pashto is one of the official spoken languages in Afghanistan [28] and the Pukhtoon community living both sides of the border of Afghanistan and Pakistan. Pashto is a language which is spoken in southern and eastern region of Afghanistan in which two studies have been conducted including Kandahar and Nangarhar. Those who speak Pashto are Pukhtoons ethnicity which is comparable with Pukhtoons in Pakistan. A population- based survey was performed to explore the prevalence of type-2 diabetes in the Pashto-speaking district of Lower Dir [29] and district Swat [30] (Khyber Pukhtoonkhuwa, a province of Pakistan). The prevalence of type-2 diabetes in Lower Dir was 11.1%, close to the average prevalence in Afghanistan found in the present analysis.

By comparing age groups, the prevalence of diabetes in 25–34 year age group was lowest (8.45%) while the highest prevalence was found in the age group 55 years and above (19.23%). People aged 55 years and over are more than twice more likely to have type-2 than those aged 25–34 years.

By stratifying regionally, the subgroup analysis showed that the prevalence of diabetes in Nangarhar (11.80%) and Kabul (11.08%) provinces was similar, and also very similar to the nearby Pakistani province of Lower Dir. Diabetes is affecting the whole of Afghanistan with the highest prevalence seen in the Kandahar province (22%) and the lowest in Balkh province (9.12%). The big difference of Kandahar compare to other provinces has not been clear. It could be due to genetics, diet or lifestyle. The cross section studies generate hypothesis for testing.

This study has several strengths as well as some limitations. To the best of our knowledge, it is the first systematic review and meta-analysis to estimate the pooled prevalence of diabetes in Afghanistan. We used a rigorous search strategy to explore eligible articles and tried to increase the comparability and quality of included articles by using robust eligibility criteria. No publication bias was found in our analysis which suggests that we did not miss any potential articles that could change the findings of this meta-analysis. The methodological quality of all included articles had a low risk bias (seven were of Good quality and three of Fair quality).

This systematic review and meta-analysis has some limitations. As expected, the meta-analyses revealed high heterogeneity in the estimated pooled prevalence. However, to deal with the problem of high heterogeneity, subgroup analyses and meta-regression were used by adding covariates to the univariate model. Due to high heterogeneity, the results of this meta-analysis should be interpreted with caution.

Another limitation of this review is that type-1 and type-2 diabetes could not distinguishable in the selected articles. Therefore, we assumed that all reported cases of diabetes were type-2, because it accounts for 90 to 95% of all diabetes cases [31].

Furthermore, this meta-analysis is based on a limited number of articles (only 6) as well as only 5 out of the 34 provinces of Afghanistan. Due to the limited number of studies in this review, only univariate meta-regression analysis is used to test the significance of each covariate instead of a multivariable meta-regression model.

## Conclusions

The results of this systematic review and meta-analysis suggest that diabetes prevalence in Afghanistan is around 12%, similar to that in neighbouring populations with similar culture and lifestyle. There are variations within the country by province and age, with those aged over 55 years having more than double the prevalence of those aged 25–34 years. There is no significant difference between males and females. Information about the prevalence of diabetes in Afghanistan is limited to 5 of the 34 provinces. Hence, more data are required about other parts of the country, with community-based diabetes testing strategies recommended to diagnose all individuals living with diabetes.

## Supplementary Information

**Additional file 1.**

## Data Availability

All relevant data is included within the manuscript file.

## Abbreviations

CI: 95% confidence interval
mg/dL: milligrams per deciliter
PRISMA: Preferred Reporting Items for Systematic Reviews and Meta-Analyses
